# Pain management research from the NIH HEAL Initiative

**DOI:** 10.3389/fpain.2023.1266783

**Published:** 2023-11-28

**Authors:** Barbara Illowsky Karp, Rebecca G. Baker

**Affiliations:** ^1^ Division of Clinical Research, National Institute of Neurological Disorders and Stroke, National Institutes of Health, Bethesda, MD, United States; ^2^ Office of the Director, National Institutes of Health, Bethesda, MD, United States

**Keywords:** pain, HEAL Initiative, research, clinical trials, NIH (National Institutes of Health)

## Abstract

This article presents an overview of the pain research programs within the National Institutes of Health (NIH) Helping to End Addiction Long-term® Initiative, or NIH HEAL Initiative®. Launched in 2018 to address the opioid crisis, the NIH HEAL Initiative supports research on addiction prevention and treatment. A key component of addiction prevention is the development of new, effective, non-addictive treatments for acute and chronic pain. HEAL's innovate research portfolio spans the spectrum from therapeutic discovery and development through clinical trials and into clinical practice.

## Introduction

Chronic pain is a significant public health concern affecting approximately 20% of adults in the United States. Approximately 4% of these individuals experience high impact pain that interferes substantially with major activities. Research and clinical experience have contributed to a continuously evolving understanding of pain. Historically, pain was initially considered to be associated with tissue damage, but current concepts incorporate the appreciation of pain as a biopsychosocial phenomenon (1). The International Association for the Study of Pain (IASP) updated its definition of pain in 2020 (2). The IASP broadly classifies pain into (i) nociceptive (pain arising from actual or threatened damage to non-neural tissue, due to activation of nociceptors), (ii) neuropathic (pain arising as a direct consequence of a lesion or disease affecting somatosensory systems), and (iii) nociplastic pain (pain arising from altered nociception despite no clear evidence of actual or threatened tissue damage or lesion of the somatosensory system). The complex and multidimensional nature of pain requires a similarly complex multifaceted approach to treatment.

The National Institutes of Health (NIH) Helping to End Addiction Long-term® Initiative, or NIH HEAL Initiative®, was launched in 2018 during a critical period in which the intersection of the opioid crisis and recognition of pain as a major public health challenge indicated that developing new options for pain management was a crucial element of fighting the opioid crisis. The HEAL Initiative applies a scientific approach to fund research to develop and implement non-addictive, effective pain mitigation strategies.

The nation's focus on pain management as a public health strategy dates back to the passage of the Patient Protection and Affordable Care Act (ACA) of 2010. This law identified the need for pain research and treatment and commissioned the National Academy of Medicine (then the Institute of Medicine, or IOM) to hold a conference to “increase the recognition of pain as a significant public health problem in the United States.” The resulting conference and 2011 IOM study committee report “Relieving Pain in America: A Blueprint for Transforming Prevention, Care, Education, and Research” identified the need for a cultural shift in how pain is understood, prevented, and treated. To enhance research efforts and to promote collaboration in pain research and treatment, the ACA also established the Interagency Pain Research Coordinating Committee (IPRCC), which includes representatives from government agencies, non-governmental research entities, healthcare organizations, providers, and patient communities. In response to the IOM report, NIH and the IPRCC analyzed the existing federal pain research portfolio, convened expert panels of working groups and consultants, and developed the National Pain Strategy (NPS) in 2016 and the National Pain Research Strategy (NPRS) in 2017. These initiatives identified gaps in pain care and research and resulted in two reports outlining new directions for pain research (3, 4). The NPS centered its recommendations around six main themes identified in the IOM report (Table 1). These reports also articulated the need to address pain across time, from prevention to acute pain and the transition to chronic pain. The reports also called for a strategy to recognize and address the heterogeneity of pain populations and pain conditions through research.

**Table 1:** Main themes for pain care and research identified by the Institute of Medicine (IOM).

1. Population research: To foster transformation, granular epidemiologic and demographic data on pain is needed, especially for minority populations and underserved communities. 2. Prevention and care: Prevention should focus on environments where injuries most often occur, such as the workplace and in populations at increased risk of developing chronic pain. 3. Addressing disparities in pain-related care: Identifying and mitigating bias and stigmatization in minority and underserved communities will help minimize disparities in pain experience and pain care. 4. Service delivery and payment: Innovative models of pain care delivery and mechanisms for reimbursement are needed. 5. Professional education: Primary care practitioners, who often lack specific training in pain management, frequently provide the first line of pain treatment. Professional organizations and pain specialists should develop materials and opportunities for pain management education for healthcare professionals. 6. Public education about pain, pain prevention and pain treatment: The scope of educational activities needs to extend to patients and the public. Patients must be partners in pain care endeavors and self-management methodologies should be incorporated as a part of a comprehensive, individualized approach.

## Addressing the opioid crisis requires new pain management strategies

Established in 2018 when the U.S. Congress added $500 M to the NIH base appropriation, the NIH HEAL Initiative consulted a broad range of partners including other government agencies (the Centers for Disease Control and Prevention, the Food and Drug Administration, the Drug Enforcement Administration), as well as academic centers, the private sector, and community patient advocates, to identify needs and opportunities and to develop “an innovative, action-oriented research plan for HEAL,” focused on improving treatments for opioid misuse and addiction and for advancing strategies for pain management (5). With the participation of many NIH Institutes and Centers, HEAL programs related to opioids and pain are spearheaded by the National Institute on Drug Abuse (NIDA) and the National Institute of Neurological Disorders and Stroke (NINDS), respectively.

Recognizing pain as an entity apart from the distinct diseases and conditions that cause it, the nation and medical community faced a significant challenge in the increasing use of opioids. The increase in opioid use over the past few decades can be attributed, to some extent, to concerns regarding the inadequate recognition and treatment of pain, which were further amplified by the influence and marketing of pharmaceutical companies, as well as the development and spread of potent synthetic opioids. Opioids prescribed for pain often provided a gateway to use of heroin as a cheaper and more accessible option and to avoid withdrawal. According to the 2020 National Survey on Drug Use and Health, 2.7 million adults in the United States have an opioid overuse disorder. As the nation ages, the prevalence of chronic pain is likely to rise, which may drive a further rise in opioid use.

The COVID-19 pandemic brought additional challenges (6). During the pandemic, the use of opioids, stimulants, and combinations of drugs rose with a concomitant increase in overdose deaths, which now exceed 100,000 per year (7). People living with pain faced many challenges, particularly early in the pandemic when in-person healthcare visits, procedures, and treatment were disrupted, interfering with implementation and continuity of care (8).

## The NIH HEAL Initiative

Cross-cutting, overarching priorities for all HEAL pain management research efforts include patient and community engagement, data harmonization and sharing, a focus on health inequities that affect pain diagnosis and treatment, and the education and training of new researchers. Community engagement assures that the needs and perspectives of people living with pain, their family members, caregivers, and healthcare providers are identified and considered. The HEAL common data elements (CDEs) are sets of required and optional CDEs with specific variables and their method of ascertainment across nine key pain-relevant domains (Table 2). The HEAL CDEs are collected in all HEAL clinical studies. As well as providing important data on symptoms that often co-occur with pain as a biopsychosocial condition, the HEAL CDE program promotes consistency and interoperability within and between HEAL programs. The HEAL data ecosystem connects the HEAL community, enabling HEAL data to be searched, analyzed, and used to make new discoveries. By empowering researchers to make their HEAL-generated data FAIR (findable, accessible, interoperable, and reusable), the HEAL data ecosystem (9) promotes data sharing. The HEAL platform, a secure, user-friendly, cloud-based search interface, aims to transform research data, findings, and publications into a virtual, annotated, searchable catalog where datasets and findings from different studies can be analyzed, compared, and combined. HEAL data are stored in various NIH-funded or other data repositories that meet appropriate standards for data security and privacy. The data generated by HEAL programs are thus available for trans-initiative analysis and meta-analysis, promoting compliance with both the NIH and HEAL data sharing policies (10). A suite of HEAL programs address systemic inequities affecting individuals and specific populations with health disparities affecting pain management. Finally, HEAL addresses the need to enhance the pain research workforce through its funding opportunities for mentorship and training of the next generation of pain researchers, with attention to promoting a diverse workforce.

**Table 2:** The common data element pain domains of the NIH HEAL Initiative.

• Pain intensity • Pain interference • Physical functioning/quality of life • Sleep • Pain catastrophizing • Depression • Anxiety • Global Satisfaction with treatment • Substance use screener

## HEAL pain research programs

Addressing pain while reducing the risk of addiction requires a broad, action-oriented, multifaceted approach predicated on understanding pain biology and pathophysiology and leading to science-informed new treatments. The ultimate goal of these programs is to improve the efficiency and effectiveness of clinical trials toward enabling personalized, precise care.

Preclinical and translational pain therapeutic development programs within HEAL start with target and asset discovery and validation, extend through into clinical trials, and encompass all clinical trial phases, including effectiveness research and pragmatic trials. Various pain management strategies are being tested including complementary and integrative approaches (some multimodal) and pain self-management approaches, as well as drugs and devices. HEAL preclinical research programs provide an avenue for focused study of pain mechanisms and therapeutics. These include preclinical testing platforms, animal models, human cell-based screening, tissue chips, and advanced imaging tools. HEAL studies incorporate techniques including neuroimaging, -omics, sensory testing, and psychosocial assessments to develop algorithms that will help predict the transition from acute to chronic pain and enable biomarker discovery, validation, and implementation.

HEAL pain programs are shown in Table 3, illustrating the breadth of the HEAL research portfolio. Within preclinical programs, the Discovery and Validation of Novel Targets for Safe and Effective Treatment of Pain program supports the initial discovery of biological targets for pain and searches the druggable genome and proteome related to pain perception, physiology, and pathophysiology. As examples, the PRECISION Human Pain Network seeks to understand human tissues involved in pain processing at the cellular and molecular levels and will subsequently use the data to validate human therapeutic targets. The Restoring Joint Health and Function to Reduce Pain Consortium (Re-JOIN) is generating models of joint sensory innervation in disease and across the lifespan by mapping the network of sensory nerves in the temporomandibular joint and the knee.

**Table 3:** The NIH HEAL Initiative pain programs.

Cross-cutting research • Increasing participant diversity, inclusion, and engagement in HEAL research • Training the next generation of researchers in HEAL • Levering existing and real-time opioid and pain management data • Data 2 action to prevent overdose • Stigma in pain management and opioid use disorder (OUD) • Small business programs
Preclinical and translational research in pain management • Discovery and validation of novel targets to safe and effective treatment of pain • Preclinical screening platform for pain • Translational research to advance testing of novel drugs and human cell-based screening platforms to treat pain and OUD • Development and optimization of non-addictive therapies to treat pain • Translating discoveries into effective devices to treat pain • Restoring joint health and function to reduce pain consortium (Re-JOIN)
Clinical research • The Back Pain Consortium Research Program (BACPAC) • The Pain Management Effectiveness Research network • Pragmatic and implementation studies for the management of pain to reduce opioid prescribing (PRISM) • EPPIC-Net • Integrative management of chronic pain and OUD for whole recovery (IMPOWR) • Integrated approach to pain and opioid use in hemodialysis patients • Acute to chronic pain signatures program • Advancing health equity in pain management • Discovery and validation of biomarkers, endpoints, and signatures for pain conditions

HEAL pain translational research programs provide the link connecting new target and therapeutic discovery and development to clinical trials. The Preclinical Screening Platform for Pain provides an evaluation of potential therapeutic assets in a tiered series of *in vitro* assays to screen for absence of opioid activity and abuse liability as well as for off-target and neurological side effects, followed by an evaluation in *in vivo* pain models.

Within HEAL clinical pain programs, the HEAL Discovery and Validation of Biomarkers, Endpoints, and Signatures program seeks to identify and validate biomarkers, endpoints, and signatures of various pain conditions. This program aims to deepen the understanding of cohort- and individual-level variations in pain perception, chronification, and response to treatment. The Back Pain Consortium Research Program (BACPAC) is studying a digital health platform that integrates quantitative spinal motion metrics, patient-reported outcomes, and consideration of patient preferences for treating low back pain. BACPAC has built a translational, patient-centered model to inform effective and personalized therapies for chronic low back pain.

Moving into clinical testing, the Early Phase Pain Investigation Clinical Network (EPPIC-Net) seeks novel or repurposed pain therapeutics from academic researchers and pharmaceutical companies. For accepted assets, EPPIC-Net designs and conducts phase 2 clinical trials and can test proposed biomarkers in its network that joins expert pain researchers and research centers. Applicants to this program collaborate in clinical trial development and, importantly, retain intellectual property rights to their asset. EPPIC-Net explores novel study designs, recently establishing a platform protocol providing efficient and robust testing of novel therapeutics for diabetic peripheral neuropathy. The Pragmatic and Implementation Studies for the Management of Pain to Reduce Opioid Prescribing program integrates evidence-based interventions into healthcare systems with pragmatic clinical trials and implementation science embedded in real-world settings.

## HEAL progress to date

To date, $3.2 billion has been invested in HEAL research, with more than 1,800 projects funded since its inception in 2018. HEAL has made substantial progress in this short time.

HEAL-funded studies have advanced several promising pain therapeutic candidates to investigational new drug and investigational device exemption filings with the Food and Drug Administration. HEAL investigators have patented novel targets for chronic pain, inflammatory and visceral pain, and migraine. Selected examples appear below.

• The Tailored Non-Pharmacotherapy Services for Chronic Pain: Testing Scalable and Pragmatic Approaches (RESOLVE) pragmatic trial compares the efficacy of two telehealth approaches to cognitive behavioral therapy for chronic pain. Establishing the utility of remote health approaches is particularly important to enable accessibility and inclusion of rural participants and of populations with limited ability to travel for in-person care.
• KnowPain: HEAL investigators have piloted a wrist-worn monitoring device collecting robust physiologic and kinematic measurements. In people living with chronic low back pain, they have shown that 7-day sensor data correlated strongly with self-reported outcomes of patient pain and function. SPRINT (Signature for Pain Recovery In Teens): The SPRINT program collects robust multimodality data from teens with chronic musculoskeletal pain, utilizing techniques that include brain imaging, motor and sensory evaluations, psychological functional assessments, and genetic and molecular profiling. Big data and machine learning algorithms will be developed and used to identify the biomarkers of pain persistence and pain resilience in this underserved population.
• Development and use of decision-making tools embedded into electronic health record systems. Offering various treatment options can help patients manage their own pain by selecting non-drug pain care after surgery, as well as help clinicians support that choice.
• The Hemodialysis Opioid Prescription Effort (HOPE) research study is testing various non-opioid treatment approaches in dialysis patients. This research is testing the use of a virtual pain coach during dialysis as well as administering buprenorphine for pain relief.
• Virtual reality allows patients with low back pain to experience 3D immersive environments, such as walking up a waterfall or exploring a virtual city.
• Discovery of a biomarker signature for neuropathic pain: This project has identified sub-basal corneal microneuromas in patients with neuropathic corneal pain, differentiating it from dry eye disease. Research to validate this potential biomarker is ongoing.
• Development of a living pain circuit (11): a tissue chip-based model system that can screen molecules in minutes.
• Non-invasive brain stimulation that combines independently controlled electromagnetic and ultrasonic fields provided pain and functional relief in a small study of patients experiencing pain due to carpal tunnel syndrome.

## Conclusions

HEAL pain research aims to improve the quality of life and reduce pain by developing and implementing safe and effective strategies for pain prevention and treatment that alleviate risks of opioids. This multifaceted research investment spans the investigational spectrum: from target discovery and validation to translational research to clinical trials and comparative effectiveness studies—in addition to growing and sustaining a vibrant pain research workforce. HEAL research is leading the way to precision medicine for the millions of Americans who live with pain. New or improved pharmacologic, non-pharmacologic, and integrated models of pain management are being tested and refined toward assembling a broad array of safe and effective approaches predicated on the needs of those with lived pain experience and their caregivers.

## Data Availability

The original contributions presented in the study are included in the article/Supplementary Material, further inquiries can be directed to the corresponding author.

## References

[1] Trachsel LA Munakomi S Cascella M. Pain theory. Treasure Island, FL: StatPearls (2023). 31424778

[2] Raja SN Carr DB Cohen M Finnerup NB Flor H Gibson S The revised International Association for the Study of Pain definition of pain: concepts, challenges, and compromises. Pain. (2020) 161(9):1976–82. 10.1097/j.pain.0000000000001939 32694387 PMC7680716

[3] National pain strategy: a comprehensive population health-level strategy for pain (2016). Available at: https://iprcc.nih.gov/National_Pain_Strategy/NPS_Main.htm. (Accessed March 13, 2023)

[4] IPRC Center. Federal pain research strategy (2017). https://www.iprcc.nih.gov/sites/default/files/documents/FPRS_Research_Recommendations_Final_508C.pdf (Accessed April 14, 2023)

[5] Collins FS Koroshetz WJ Volkow ND. Helping to end addiction over the long-term: the research plan for the NIH HEAL Initiative. JAMA. (2018) 320(2):129–30. 10.1001/jama.2018.8826 29896636 PMC6688196

[6] Baker RG Koroshetz WJ Volkow ND. The helping to end addiction long-term (HEAL) initiative of the National Institutes of Health. JAMA. (2021) 326(11):1005–6. 10.1001/jama.2021.13300 34436519

[7] Ahmad FB Cisewski JA Rossen LM Sutton P. Provisional drug overdose death counts (2023). Available at: https://www.cdc.gov/nchs/nvss/vsrr/drug-overdose-data.htm. (Accessed April 14, 2023)

[8] Shanthanna H Strand NH Provenzano DA Lobo CA Eldabe S Bhatia A Caring for patients with pain during the COVID-19 pandemic: consensus recommendations from an international expert panel. Anaesthesia. (2020) 75(7):935–44. 10.1111/anae.15076 32259288 PMC7262200

[9] About the HEAL data ecosystem. Available at: https://heal.nih.gov/data/heal-data-ecosystem. (Accessed April 5, 2023)

[10] Data management & sharing policy overview (2023). Available at: https://sharing.nih.gov/data-management-and-sharing-policy/about-data-management-and-sharing-policies/data-management-and-sharing-policy-overview. (Accessed April 14, 2023)

[11] Pollard KJ Bowser DA Anderson WA Meselhe M Moore MJ. Morphine-sensitive synaptic transmission emerges in embryonic rat microphysiological model of lower afferent nociceptive signaling. Sci Adv. (2021) 7(35):eabj2899. 10.1126/sciadv.abj2899 34452921 PMC8397270

